# Effect of Nanoclay Modification and Environmental Exposure on the Mechanical and Surface Properties of Polyester Composites

**DOI:** 10.3390/ijms27146199

**Published:** 2026-07-11

**Authors:** Dominik Stępka, Magdalena Bańkosz, Karina Rusin-Żurek, Dagmara Słota, Karina Niziołek, Kinga Setlak, Katarzyna Haraźna, Josef Jampilek, Agnieszka Sobczak-Kupiec

**Affiliations:** 1 Department of Materials Science, CUT Doctoral School, Faculty of Materials Engineering and Physics, Cracow University of Technology, 37 Jana Pawła II Av., 31-864 Krakow, Poland; dominik.stepka@doktorant.pk.edu.pl (D.S.); karina.rusin-zurek@pk.edu.pl (K.R.-Ż.); karina.niziolek@pk.edu.pl (K.N.); 2 Department of Materials Science, Faculty of Materials Engineering and Physics, Cracow University of Technology, 37 Jana Pawła II Av., 31-864 Krakow, Poland; magdalena.bankosz@pk.edu.pl (M.B.); kinga.setlak@pk.edu.pl (K.S.); katarzyna.harazna@pk.edu.pl (K.H.); agnieszka.sobczak-kupiec@pk.edu.pl (A.S.-K.); 3 Department of Analytical Chemistry, Faculty of Natural Sciences, Comenius University, Ilkovicova 6, 842 15 Bratislava, Slovakia; 4 Department of Chemical Biology, Faculty of Science, Palacky University, Slechtitelu 27, 779 00 Olomouc, Czech Republic

**Keywords:** composites, polyester resin, montmorillonite, nanoclay

## Abstract

This article presents the results of a study investigating the influence of surface-modified montmorillonite nanoclay and environmental exposure on the mechanical and surface properties of composites based on orthophthalic unsaturated polyester resin. A series of samples containing 0.02 to 0.1 wt.% of nanoclay, as well as a reference sample, were prepared. Following synthesis, the samples were incubated for one week in aqueous solutions with pH values of 4, 7, and 9. Mechanical testing included tensile strength, flexural strength, impact resistance, and surface roughness (Ra). Additionally, fracture surface analysis was performed using scanning electron microscopy (SEM) and digital optical microscopy. The effect of the additive on hardness was also assessed, and Fourier transform infrared (FT-IR) spectroscopy was carried out. The results indicated that nanoclay addition increased the material’s stiffness (Young’s modulus), although higher filler concentrations led to decreased impact strength and flexural performance, likely due to particle agglomeration. Incubation in chemically aggressive environments caused mechanical degradation and significant increases in surface roughness, especially after exposure to neutral and alkaline media. Microscopic observations confirmed the presence of microstructural changes and nanoclay agglomerates in selected samples. The findings confirm that the effectiveness of nanoclay modification depends not only on concentration but also on dispersion quality and the environmental conditions to which the material is exposed.

## 1. Introduction

Polymer composites reinforced with nanofillers, especially nanoclays, have been the subject of intensive research in materials engineering for many years. Their growing importance stems from their potential to improve the mechanical, thermal, and barrier properties of polymer materials [1,2,3]. A particularly notable nanoclay in this category is montmorillonite (MMT), a layered silicate mineral that exhibits a unique ability to intercalate and exfoliate within polymer matrices, resulting in significant enhancement of the physicochemical properties of the composites [4,5]. Montmorillonite’s ability to undergo intercalation and exfoliation makes its structure easily modifiable using appropriate organic agents. Such surface modification increases the compatibility of MMT with hydrophobic polymers, promoting a more homogeneous dispersion of the nanoclay within the matrix. A properly prepared mixture leads to the formation of composites with significantly improved performance parameters, such as increased stiffness, tensile strength, and flexural resistance [6,7,8].

At the same time, the effectiveness of this modification depends not only on the type and concentration of the filler used but also on the degree of its dispersion, the type of polymer matrix, and the environmental conditions under which the composite is used. In terms of material durability, it is crucial to study the effects of environmental exposure, including varying pH conditions, which may influence the chemical and physical stability of the composites [9,10,11]. Acidic and alkaline environments, in particular, can initiate degradation processes both within the polymer matrix and at the polymer–filler interface. These conditions may cause microstructural changes such as delamination, cracking, or a reduction in interfacial adhesion, which directly impact the mechanical properties of the final product [12,13].

Several studies have demonstrated that the incorporation of montmorillonite nanoclay can improve the mechanical performance of polymer composites, particularly in terms of stiffness, tensile strength, and flexural behavior [14,15]. However, the effectiveness of nanoclay reinforcement depends strongly on factors such as filler concentration, dispersion quality, and matrix–filler interactions [16,17]. While low nanoclay contents often lead to improvements in composite performance, excessive filler loading may promote particle agglomeration, resulting in stress concentration and deterioration of mechanical properties [18]. These findings indicate that the beneficial effects of nanoclay are not solely related to its presence within the polymer matrix, but also to the manner in which it is distributed and integrated into the composite structure. While nanoclay-reinforced polymer composites have been widely studied, limited attention has been paid to the behavior of polyester composites containing very low nanoclay loadings when exposed to environments of different pH. Furthermore, the combined influence of environmental exposure and nanoclay modification on both mechanical performance and surface characteristics has not been sufficiently explored. This study aims to evaluate the effect of low concentrations of montmorillonite nanoclay modification on the microstructural changes and mechanical properties of polyester resin-based composites, both before and after exposure to acidic, neutral, and alkaline environments with varying pH levels.

## 2. Results

### 2.1. Fourier Transform Infrared Spectroscopy Analysis

**Figure 1:**
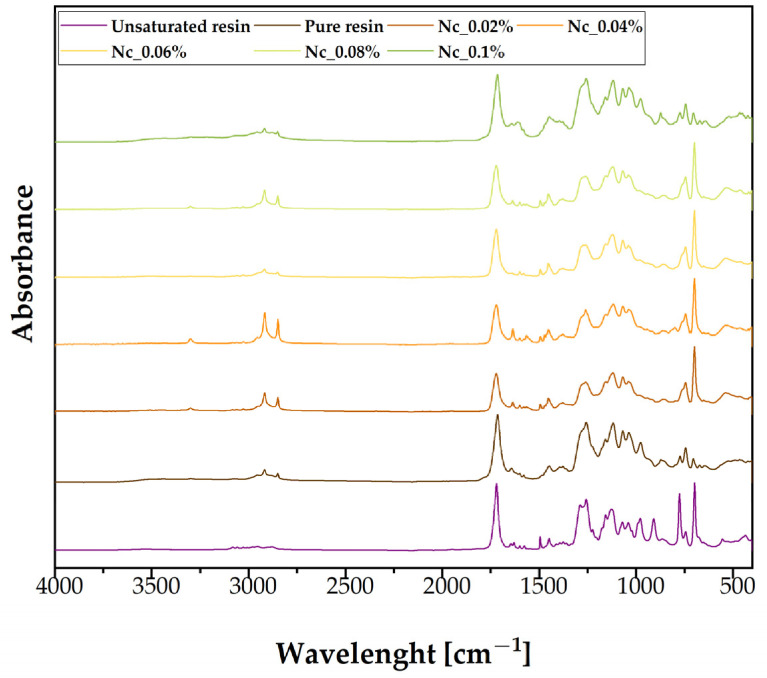
FTIR spectra of pure resin and resin/nanoclay composites with different nanoclay contents (Nc_0.02%, Nc_0.04%, Nc_0.06%, Nc_0.08%, and Nc_0.1%).

Analysis of the FTIR spectra shown in Figure 1 revealed the presence of characteristic absorption bands for both the pure resin and the nanoclay-modified (Nc) composites. The spectrum of the pure resin serves as a reference for evaluating the effect of the nanoclay additive on the material’s chemical structure.

In the spectrum of pure resin, a broad band is observed in the range of approximately 3400–3500 cm^−1^, corresponding to the stretching vibrations of the hydroxyl (O-H) groups. The presence of this signal is mainly associated with adsorbed moisture and terminal hydroxyl groups present in the structure of unsaturated polyester resin [19]. The bands appearing at approximately 2920 cm^−1^ and 2850 cm^−1^ have been attributed to the asymmetric and symmetric stretching vibrations of the C-H bonds of the aliphatic groups -CH_2_ and -CH_3_ present in the polymer chains of the resin [20]. To evaluate the curing process, the FTIR spectrum of the uncured polyester resin was compared with that of the cured resin. As shown, the curing process resulted in a decrease in the intensity of bands associated with unsaturated groups participating in crosslinking, particularly in the region around 1630–1640 cm^−1^, attributed to C=C stretching vibrations, and in the 980–910 cm^−1^ region, related to vinyl (=C-H) vibrations. In contrast, the characteristic ester carbonyl band at approximately 1720 cm^−1^ remained present after curing. These spectral changes qualitatively confirm the progress of the curing reaction and the formation of the crosslinked polyester network. Additional changes in band intensity were also observed in the 2800–3000 cm^−1^ region, corresponding to C-H stretching vibrations of aliphatic and styrene-related groups. The reduction of these bands after curing is consistent with the consumption of unsaturated species during the crosslinking process.

The most characteristic band for polyester resin is an intense band in the 1720–1730 cm^−1^ range, corresponding to the stretching vibrations of ester carbonyl groups (C=O), typical of unsaturated polyester resins [21]. The bands observed in the 1600–1510 cm^−1^ range are associated with skeletal vibrations of C=C bonds in aromatic rings derived from the orthophthalic structure and styrene residues [20]. Signals in the 1450–1380 cm^−1^ range correspond to deformational vibrations of the -CH_2_ and -CH_3_ groups, while bands at 1260–1120 cm^−1^ have been attributed to stretching vibrations of C-O-C ester bonds. Additionally, bands in the 980–700 cm^−1^ range are associated with out-of-plane vibrations of =C-H bonds and aromatic styrene groups present in the resin [22].

In the case of composites containing nanoclay (Nc_0.02%, Nc_0.04%, Nc_0.06%, Nc_0.08%, and Nc_0.1%), changes in the intensity of selected bands and the appearance of signals characteristic of layered aluminosilicate are observed. As the nanoclay content increases, the intensity of the band in the 1030–1000 cm^−1^ range increases, corresponding to the stretching vibrations of the Si-O-Si and Si-O-Al bonds present in the montmorillonite structure [23]. This effect is particularly evident for the Nc_0.08% and Nc_0.1% samples, confirming the presence of a silicate phase in the composite material.

Additionally, in samples containing nanoclay, an increase in the intensity of the band around 3400 cm^−1^ is observed, attributed to hydroxyl groups present on the surface of the nanoclay and water molecules bound between the montmorillonite layers. The band around 1640 cm^−1^ corresponds to the deformational vibrations of H-O-H in adsorbed water. In the 520–470 cm^−1^ region, additional signals appear associated with the deformational vibrations of Si-O-Al and Si-O-Mg bonds, characteristic of the montmorillonite aluminosilicate structure [24].

The FTIR results indicate that the incorporation of nanoclay did not lead to significant changes in the characteristic absorption bands of the polyester resin, suggesting that no new chemical bonds were formed between the matrix and the filler. The appearance and increasing intensity of the Si-O-Si and Si-O-Al bands confirmed the presence of montmorillonite in the composites. Therefore, the changes observed in the mechanical properties are likely associated with physical reinforcement mechanisms, including filler dispersion and matrix–filler interactions, rather than with chemical modification of the polymer network. This interpretation is consistent with the increase in stiffness observed for selected nanoclay concentrations and with the deterioration of some mechanical properties at higher filler contents, which may be attributed to non-uniform filler distribution.

### 2.2. X-Ray Diffraction Analysis

**Figure 2:**
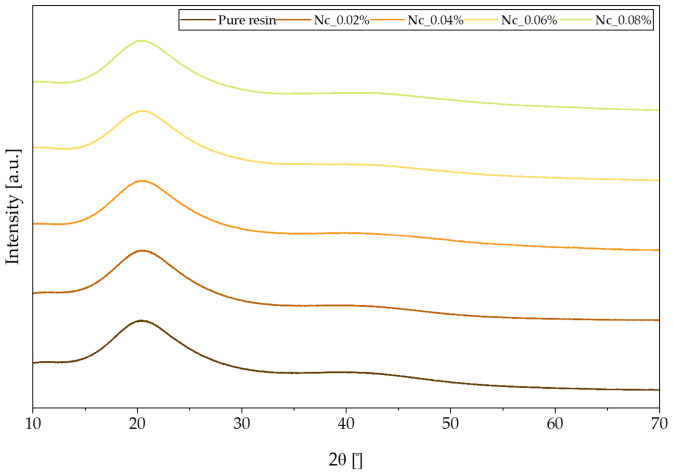
Diffractograms of pure resin and resin/nanoclay composites with different nanoclay contents (Nc_0.02%, Nc_0.04%, Nc_0.06%, Nc_0.08%, and Nc_0.1%).

The XRD patterns of the pure polyester resin and nanoclay-modified composites are presented in Figure 2. All samples exhibited a broad diffraction halo centered at approximately 20–22° (2θ), which is characteristic of the predominantly amorphous structure of the polyester resin matrix. The incorporation of nanoclay at concentrations ranging from 0.02 to 0.08 wt.% did not result in the appearance of additional diffraction peaks or any noticeable shift in the broad amorphous halo. The diffraction profiles of all modified samples remained similar to that of the unfilled resin, indicating that the amorphous nature of the matrix was preserved after nanoclay incorporation. A gradual increase in the intensity of the broad diffraction halo was observed with increasing nanoclay content. The maximum remained located within the same 2θ range for all compositions, suggesting that the addition of nanoclay did not significantly alter the overall structural arrangement of the polyester matrix. No distinct reflections associated with crystalline nanoclay phases were detected within the investigated concentration range.

The absence of additional diffraction peaks and the unchanged position of the amorphous halo suggest that nanoclay incorporation at the investigated concentrations did not induce detectable changes in the molecular packing or structural organization of the polyester matrix. These observations are consistent with the FTIR results, which also indicated that nanoclay addition did not lead to substantial modifications in the chemical structure of the cured resin. Therefore, the variations observed in the mechanical properties are more likely associated with microstructural factors, such as filler dispersion, local agglomeration, and matrix–filler interactions, rather than with significant changes in the molecular structure of the polymer network.

### 2.3. Tensile Strength Tests

**Figure 3:**
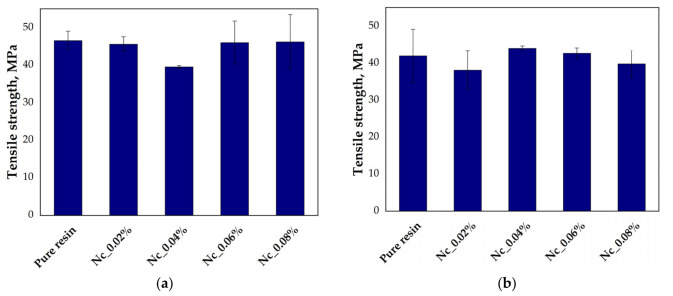

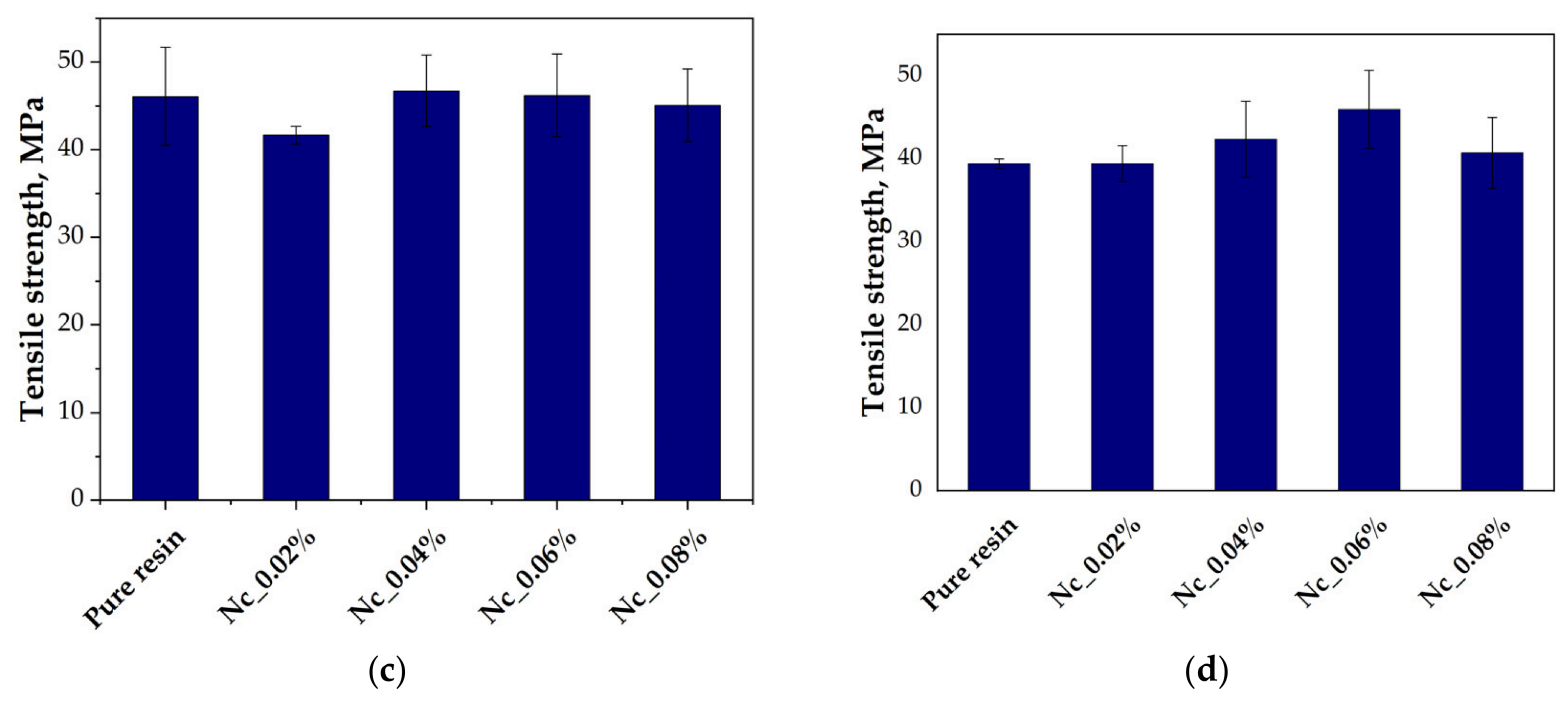
Tensile strength of samples before incubation (**a**) and after incubation at pH 4 (**b**), pH 7 (**c**), and pH 9 (**d**).

The results of the tensile strength tests are presented in Figure 3 for both the unmodified and nanoclay-modified samples. Measurements were carried out before incubation and after exposure to environments with pH levels of 4, 7, and 9.

The analysis of tensile strength results allows for an evaluation of the effects of nanoclay content and exposure to different pH environments on the mechanical properties of polyester composites. For samples tested prior to incubation, the addition of nanoclay at concentrations of 0.02%, 0.06%, and 0.08% by weight did not lead to significant changes in tensile strength compared to the pure resin, which exhibited a value of 46 MPa. These values were within the range of standard deviation, suggesting a stable influence of low nanoclay concentrations on mechanical behavior. However, at a 0.04% loading, a noticeable decrease to 39 MPa was observed, which may indicate issues such as poor dispersion of the filler or unfavorable interfacial interactions.

After incubation in an acidic environment (pH 4), a general decline in tensile strength was observed across all samples, likely due to the degradation of the polymer network caused by H^+^ ion activity. The strength of the pure resin decreased to 42 MPa, with the lowest value recorded for the 0.02% nanoclay sample (38.11 MPa). In a neutral environment (pH 7), the mechanical performance of the composites remained relatively unchanged compared to the initial values, indicating minimal degradation. The tensile strength of the pure resin was 46 MPa, while the nanoclay-modified samples ranged from 42 MPa (0.02%) to 47 MPa (0.04%). These results confirm the good chemical resistance of the materials in neutral conditions and highlight the potential stabilizing role of nanoclay in aqueous environments. Incubation in an alkaline solution (pH 9) led to a clear reduction in strength for the pure resin, dropping to 39 MPa, suggesting a higher susceptibility to alkaline degradation. Among the nanoclay-reinforced samples, results varied. The highest value was recorded for the 0.06% sample (46 MPa), indicating that an optimal filler concentration may mitigate degradation in basic conditions. Conversely, the strength of the 0.08% sample decreased to 41 MPa, suggesting that exceeding the optimal nanoclay loading may lead to agglomeration and reduced performance.

### 2.4. Stiffness

**Figure 4:**
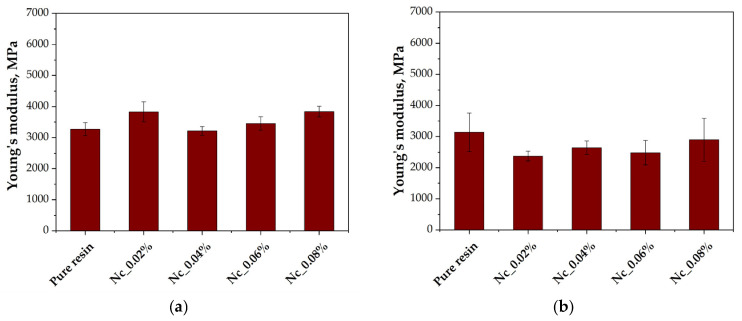

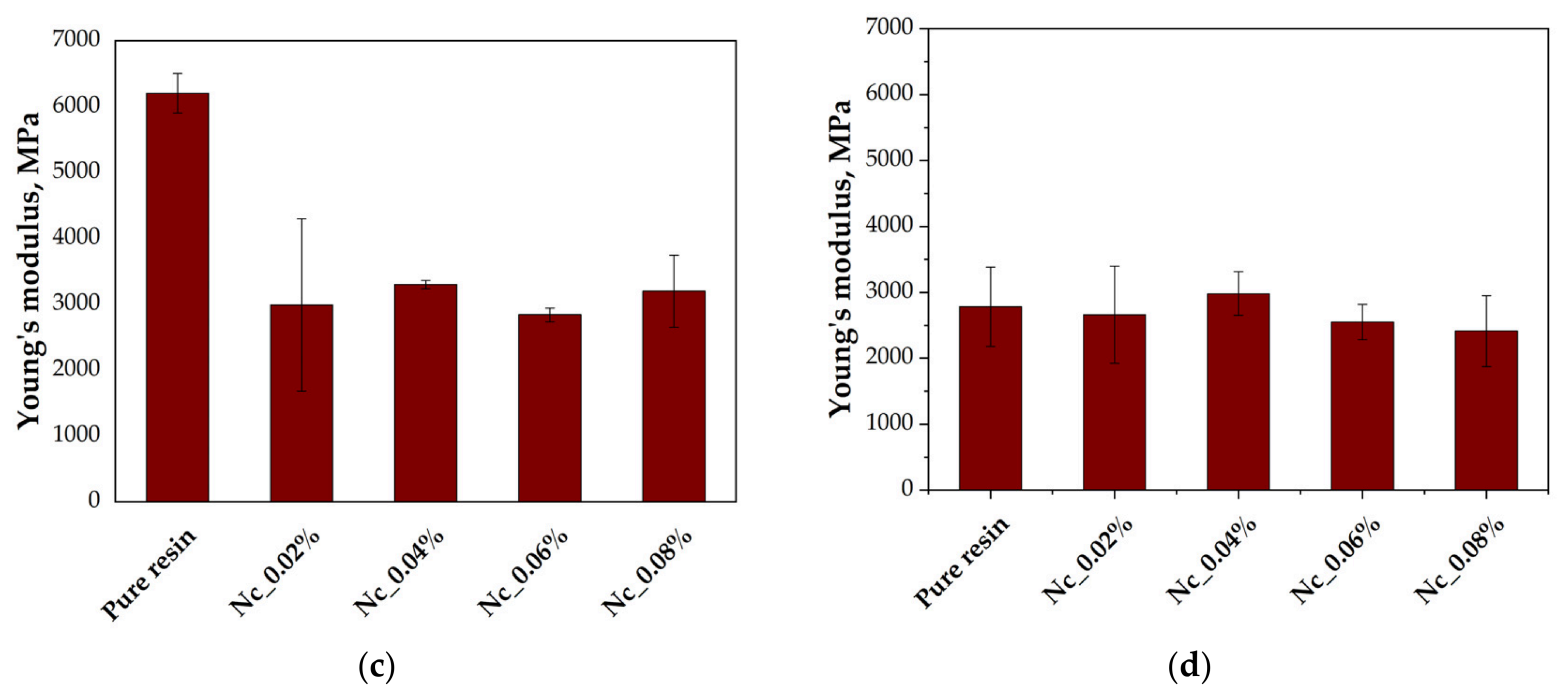
Young’s modulus of samples before incubation (**a**) and after incubation at pH 4 (**b**), pH 7 (**c**), and pH 9 (**d**).

The stiffness of the samples was evaluated based on Young’s modulus. The results are presented in Figure 4, showing how nanoclay content and incubation conditions influenced the elastic behavior of the composites.

The Young’s modulus results provide further insight into the stiffness of the composite materials and the influence of nanoclay content combined with environmental exposure. Prior to incubation, all nanoclay-modified samples, except the one containing 0.04%, exhibited increased stiffness compared to the unmodified resin, which had a modulus of 3269 MPa. The most notable increases were observed at 0.02% and 0.08% nanoclay concentrations, suggesting an effective load transfer and reinforcing effect at these filler levels. However, the sample containing 0.04 wt.% nanoclay exhibited a lower modulus than the reference material. Although FTIR analysis confirmed the progress of the curing reaction in the investigated system, the mechanism responsible for the reduced stiffness cannot be conclusively identified based on the available results. Possible contributing factors include local filler agglomeration, non-uniform filler distribution, or matrix–filler interactions that may have affected stress transfer within the composite. Following incubation in an acidic environment (pH 4), a general decrease in stiffness was observed across all samples, regardless of nanoclay concentration. This decline suggests that acidic conditions adversely affect the structural integrity of the composites, likely through chemical degradation of the polymer matrix. The reduction in Young’s modulus may also be linked to weakened interfacial bonding and partial hydrolysis of ester linkages within the polyester structure, which can compromise the material’s rigidity. In a neutral environment (pH 7), the mechanical response was more varied. Some samples demonstrated minor changes in stiffness, while others showed more significant shifts. Notably, the pure resin exhibited a marked increase in modulus, which could be attributed to post-curing or further crosslinking occurring during incubation. These mixed results indicate that the structural stability of the composites under neutral conditions is strongly influenced by the nanoclay dispersion quality and the interaction between the filler and matrix. In alkaline conditions (pH 9), a consistent reduction in stiffness was observed, suggesting partial degradation of the polyester network. The presence of OH^−^ ions can lead to base-catalyzed hydrolysis of ester bonds, a known mechanism of polymer degradation in such environments. While nanoclay may offer some degree of protection by acting as a barrier, its effectiveness appears limited at higher pH levels. This may be due to filler agglomeration or insufficient matrix–filler interaction, particularly at elevated nanoclay concentrations.

### 2.5. Flexural Strength Tests

**Figure 5:**
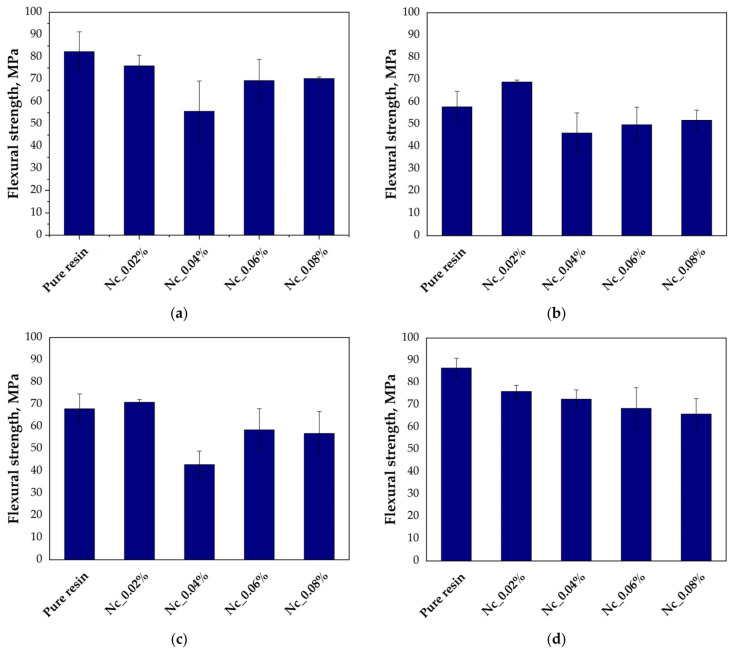
Flexural strength of samples before incubation (**a**) and after incubation at pH 4 (**b**), pH 7 (**c**), and pH 9 (**d**).

Flexural strength tests were conducted to further evaluate the mechanical response of the composites under three-point bending stress. The results are presented in Figure 5 and reflect the combined effect of nanoclay content and incubation environment on the structural performance of the materials.

Prior to incubation, the reference sample of pure polyester resin exhibited the highest flexural strength at 82.5 MPa. The incorporation of nanoclay generally led to a decrease in bending strength, with the most significant reduction observed at 0.04% content (56 MPa), possibly indicating insufficient dispersion or negative interfacial effects at this concentration. Samples with 0.02%, 0.06%, and 0.08% nanoclay also showed lower values than the unmodified resin, although with less pronounced differences. This trend may suggest that, unlike in tensile loading, the nanoclay did not reinforce the composite effectively under three-point bending stress, at least in the initial state. After acidic incubation (pH 4), a clear reduction in flexural strength was noted in most samples, including the unmodified resin, indicating the degrading effect of acidic conditions on structural performance. Interestingly, some nanoclay-modified samples showed relatively improved resistance compared to the reference resin, suggesting that the presence of nanoclay may offer partial protection against acid-induced degradation. However, overall values remained lower than in the pre-incubation state, confirming the vulnerability of the polymer network in acidic environments. Under neutral conditions (pH 7), the mechanical response was more variable. The pure resin showed a moderate decrease compared to the initial value, while some nanoclay-containing samples achieved comparable or slightly improved strength. This suggests that neutral pH conditions did not significantly compromise structural integrity and that nanoclay presence may help maintain flexural performance in such environments. However, large standard deviations in some samples may indicate heterogeneity in filler distribution or localized degradation. Incubation in alkaline conditions (pH 9) resulted in improved flexural strength for all samples, including the unmodified resin, which reached a maximum value of 87 MPa. This may point to post-curing effects or temporary stiffening due to interactions between the alkaline environment and the polymer matrix. Nanoclay-modified samples also showed enhanced performance compared to acidic or neutral conditions, although values remained slightly below those of the pure resin. These results suggest a more complex interaction between the composite structure and the chemical environment, where mild alkaline exposure may temporarily enhance certain mechanical properties.

### 2.6. Flexural Modulus

**Figure 6:**
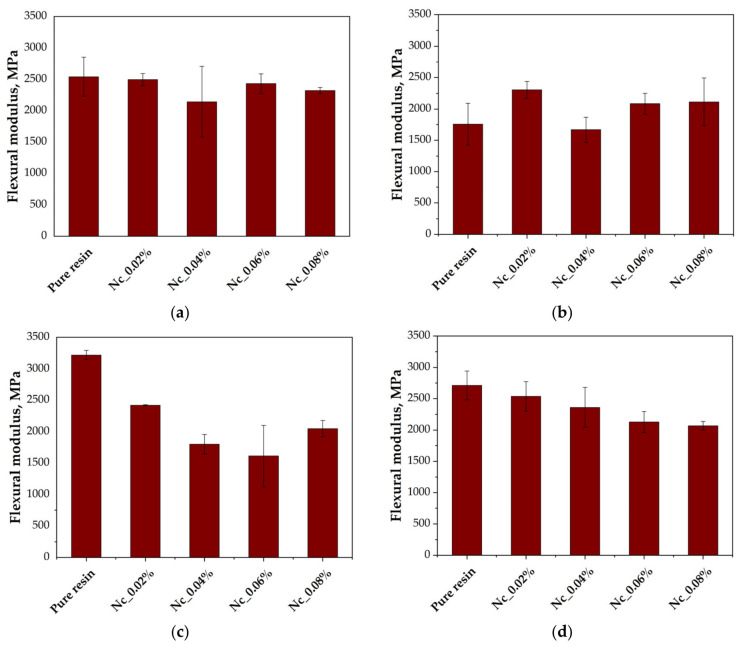
Flexural modulus of samples before incubation (**a**) and after incubation at pH 4 (**b**), pH 7 (**c**), and pH 9 (**d**).

To complete the characterization of the mechanical properties of the composites, the flexural modulus was also measured. The results are shown in Figure 6, which illustrates the effect of nanoclay addition and incubation in different pH environments on the stiffness of the materials under flexural loading.

The analysis of flexural modulus provides insight into how the stiffness of the composites is influenced by the presence of nanoclay and exposure to different chemical environments. In the unaged state, the pure resin sample exhibited the highest stiffness, while the addition of nanoclay, regardless of concentration, generally led to a moderate decrease in modulus. This reduction may be attributed to the limited reinforcing effect of the nanofiller under bending loads or to suboptimal dispersion of nanoparticles within the polymer matrix. When exposed to different pH conditions, the materials displayed distinct behaviors, indicating that environmental factors significantly affect mechanical performance, though not uniformly. In acidic conditions, a general decline in flexural modulus was observed, suggesting structural degradation of the polymer network. However, some nanoclay-modified composites demonstrated relatively higher stability compared to the reference material, possibly due to a protective effect of the filler, which may hinder the diffusion of reactive species into the matrix.

In contrast, incubation in neutral and alkaline environments resulted in more varied responses that cannot be solely attributed to pH. The pure resin showed a marked increase in stiffness under neutral conditions, potentially due to secondary crosslinking or structural tightening during prolonged exposure to water. Such effects were not evident in nanoclay-containing samples, many of which exhibited reduced stiffness. Under alkaline conditions, the results were more consistent, with smaller differences between samples and relatively stable modulus values. This suggests that basic environments did not drastically compromise the material structure under bending stress. Overall, the results indicate that nanoclay modification does not consistently enhance flexural stiffness. The effectiveness of the filler appears to depend on factors such as dispersion quality, loading conditions, and environmental exposure. In some cases, the presence of nanoclay may even contribute to a decrease in stiffness, highlighting the complex nature of its interaction with the polymer matrix.

### 2.7. Impact Strength

**Figure 7:**
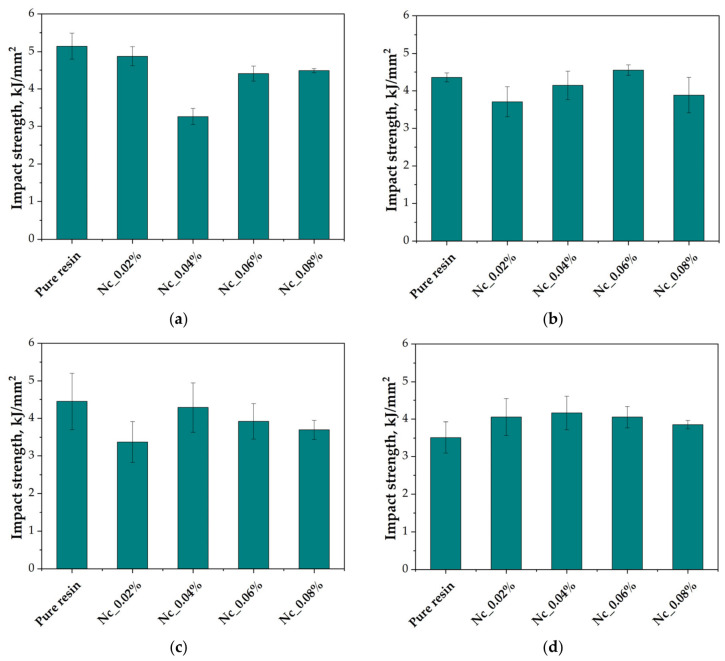
Impact strength of samples before incubation (**a**) and after incubation at pH 4 (**b**), pH 7 (**c**), and pH 9 (**d**).

The Charpy impact strength values of the composites, measured before and after incubation under different pH conditions, are presented in Figure 7.

The impact resistance of the tested composites appears to be sensitive both to the nanoclay content and to environmental exposure. In the unmodified state, the pure resin sample exhibited the highest energy absorption upon fracture, which is consistent with the behavior of a ductile, homogeneous material. The introduction of nanoclay, particularly at 0.04%, resulted in a noticeable reduction in impact strength, likely due to the formation of localized stress concentrations or microcracks near the filler particles. This highlights that while nanofillers may reinforce the structure under static loads, they can also introduce discontinuities that compromise the material’s resistance to dynamic stresses. Following incubation in different pH environments, no consistent trend in impact strength was observed, suggesting a complex interplay between material degradation and structural response. In many cases, a reduction in impact resistance was recorded, particularly for the pure resin incubated under alkaline conditions, which may be attributed to the breakdown of ester linkages and embrittlement of the matrix. Conversely, some nanoclay-reinforced samples maintained relatively stable or even slightly improved impact strength, indicating a possible role of the filler in dissipating fracture energy or mitigating crack propagation.

However, no clear correlation between nanoclay concentration and impact strength could be established. The effect appears to be mediated by the dispersion quality of the filler and the overall structural response of the composite to environmental stressors. These findings suggest that impact resistance is especially sensitive to subtle microstructural changes, and that the matrix–filler interactions can both weaken or enhance the dynamic toughness of the material depending on the chemical context.

### 2.8. Hardness Measurement

**Figure 8:**
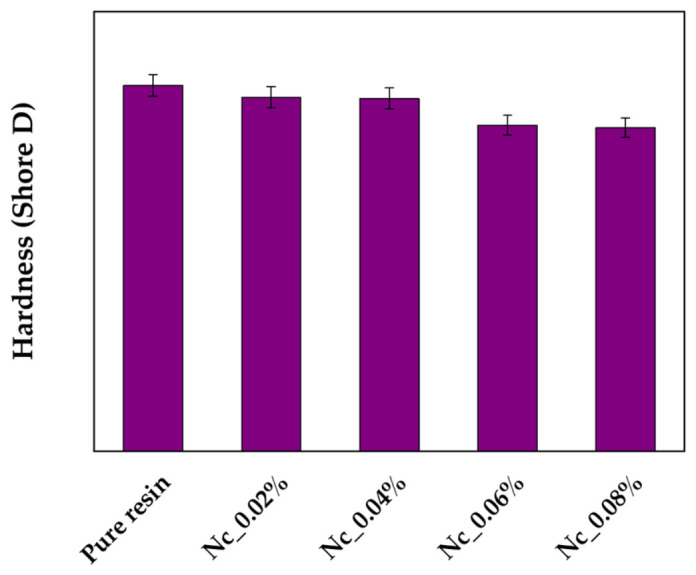
Assessment of the effect of nanoclay additive on resin hardness.

Figure 8 presents the effect of nanoclay addition on the hardness of the tested materials. The pure resin exhibited the highest hardness among all analyzed samples. The incorporation of nanoclay resulted in a gradual decrease in Shore D hardness. For low nanoclay contents (0.02% and 0.04%), the reduction was slight, whereas higher nanoclay loadings (0.06% and 0.08%) led to a more pronounced decrease in hardness.

The reduction in hardness may be attributed to the hindering effect of nanoclay particles on the resin crosslinking process, as well as to the local formation of agglomerates acting as structural defects within the polymer matrix. As a consequence, the material becomes slightly less resistant to local surface deformation, as determined by the Shore D hardness test.

### 2.9. Microscopy Analysis

An additional insight into the structural changes in the composites was obtained through scanning electron microscopy (SEM). Table 1 presents a comparative set of SEM fracture surface images taken after tensile testing, for both unincubated and incubated samples. To further characterize the influence of environmental exposure on surface morphology, Table 2 includes representative surface images acquired using a high-resolution digital microscope (Keyence VKX-7000), showing topographical features of the composites before and after incubation under different pH conditions.

The fracture surface morphology observed via scanning electron microscopy (SEM), as presented in Table 1, provides a general overview of the effects of incubation and nanoclay modification on the internal structure of the tested composites. For samples exposed to different pH environments, visible changes in fracture surface texture were noted, which may reflect chemical interactions between the matrix and the incubation medium. In nanoclay-modified samples, several images reveal the presence of irregularly distributed particle clusters, which may indicate localized agglomeration of the filler. Such microstructural heterogeneities could partially explain the variation observed in mechanical performance, particularly in properties such as flexural strength and impact resistance. The presence of particle clusters may act as local stress concentration sites, facilitating crack initiation under mechanical loading. The more heterogeneous fracture morphology observed for selected nanoclay-modified samples suggests that crack propagation may be influenced by local variations in filler distribution. Conversely, samples exhibiting more uniform fracture surfaces generally showed better mechanical performance, indicating a more effective stress transfer between the matrix and the dispersed nanoclay particles. Smoother and more uniform fracture surfaces were generally observed in unincubated specimens, whereas post-incubation samples, especially those exposed to acidic and alkaline conditions, exhibited rougher and more irregular textures, potentially suggesting ongoing degradation of the polymer matrix. Furthermore, the increased fracture surface roughness observed after incubation may indicate the development of microstructural defects, such as microvoids and weak interfacial regions, which can contribute to premature crack propagation. These observations are consistent with the reduction in selected mechanical properties recorded after environmental exposure and suggest that matrix degradation and interfacial deterioration play an important role in the fracture behavior of the investigated composites.

The surface images obtained using the Keyence digital microscope, presented in Table 2, did not reveal any distinct morphological differences between the samples or between the pre- and post-incubation states. Therefore, surface analysis was complemented by a quantitative assessment of roughness based on the Ra parameter. Although qualitative differences were limited, microscopic observations revealed a tendency toward a less homogeneous surface appearance after incubation, particularly for samples exposed to neutral and alkaline environments. These subtle changes suggest the occurrence of surface deterioration processes that are more effectively captured through quantitative roughness measurements than by visual inspection alone. The results of this analysis are summarized in Table 3.

The average surface roughness values (Ra), summarized in Table 3, clearly indicate that incubation in environments of varying pH resulted in moderate changes in the surface topography of the investigated composites. Although variations in roughness were observed after environmental exposure, the magnitude of these changes remained relatively limited, suggesting that the surface integrity of the polyester matrix was generally preserved during the incubation period. The response of the materials depended on both the pH of the environment and the nanoclay content. In some cases, particularly for composites containing 0.02 wt.% and 0.08 wt.% nanoclay, an increase in roughness was observed after incubation, whereas other formulations exhibited only minor changes relative to the initial state. No clear monotonic relationship between nanoclay concentration and surface roughness was identified. This observation suggests that local microstructural factors, including filler dispersion and matrix–filler interactions, may play a more important role than filler concentration alone. The roughness results are consistent with the microscopy observations, which revealed only limited changes in surface morphology following incubation. The combined microscopy and roughness analyses therefore support the hypothesis that environmental exposure progressively alters the surface and near-surface regions of the composites, which may contribute to the deterioration of their mechanical performance.

## 3. Discussion

The mechanical characterization of nanoclay-reinforced polyester composites revealed that the effect of this type of filler is highly dependent on both the loading conditions and environmental factors. It should be noted that the present study focused primarily on the relationship between mechanical performance, surface characteristics, and environmental exposure. Therefore, the discussion of the underlying mechanisms is based on the observed experimental trends together with the available FTIR, XRD, and microscopy results. In the non-incubated state, the incorporation of nanoclay led to a noticeable increase in Young’s modulus in most formulations, indicating enhanced stiffness and improved elastic resistance. This behavior can be attributed to more effective stress transfer between the polymer matrix and well-dispersed nanofiller particles. Similar effects were reported by Shettar et al., who observed a comparable improvement in stiffness in epoxy-based nanocomposites with low nanoclay content [25]. However, when evaluating more complex mechanical responses, such as flexural strength and impact resistance, the results showed less consistent behavior. In particular, selected formulations (e.g., with 0.04% nanoclay) demonstrated reductions in mechanical performance, which may be linked to filler agglomeration and the formation of microstructural discontinuities that act as stress concentrators. Ghaffari et al. noted comparable observations in PMMA-based nanocomposites, where increasing nanoclay content led to a deterioration of flexural properties due to the loss of homogeneity and poor particle dispersion [26]. Environmental exposure introduced an additional layer of complexity. Samples incubated in acidic conditions (pH 4) experienced clear degradation in tensile and flexural properties. This is likely associated with hydrolysis of ester bonds within the polyester matrix, weakening the polymer network and decreasing its load-bearing capacity. These effects are in line with previous findings related to acid-induced degradation in polyester and epoxy composites [27,28,29]. Under neutral conditions (pH 7), the mechanical response was more variable, with some samples exhibiting increased stiffness. Although the exact mechanism cannot be conclusively identified based on the available results, this behavior may be associated with changes occurring during environmental exposure and their interaction with the composite microstructure. Interestingly, such effects were not consistently observed across nanoclay-containing samples, suggesting that the quality of filler dispersion may significantly influence how the material responds to aqueous environments. Alkaline incubation (pH 9) produced more stable results. While the unmodified polyester resin showed evidence of degradation, likely due to base-catalyzed hydrolysis of ester linkages, some nanoclay-reinforced samples maintained their mechanical integrity. This may be due to the filler’s ability to act as a physical barrier against hydroxide ion penetration. Nevertheless, the effectiveness of this mechanism appears to be concentration-dependent and influenced by the homogeneity of the composite microstructure. The overall results are consistent with the existing literature. Patel et al. reported that a 5 wt% nanoclay loading in bamboo fiber-reinforced polyester composites led to mechanical enhancement, but further increases in nanoclay content resulted in reduced performance due to particle agglomeration [30]. FTIR and XRD analyses performed in the revised study indicated that nanoclay incorporation did not result in substantial changes in the chemical structure or overall structural organization of the polyester matrix. Consequently, the observed differences in mechanical performance are more likely related to microstructural factors, including filler dispersion quality, local agglomeration, and matrix–filler interactions, rather than to major changes in the polymer network itself. Obtained results reinforce the conclusion that nanoclay effectiveness is not a simple function of its concentration. Rather, its impact is determined by a combination of dispersion quality, matrix–filler interactions, and environmental stressors.

## 4. Materials and Methods

### 4.1. Materials

The primary matrix material used in this study was an orthophthalic unsaturated polyester resin, commonly employed in the production of pipes and tanks via the Continuous Filament Winding (CFR) process. This resin is known for its viscosity of approximately 205 mPas at 23 °C, monomer content of around 44%, and flexural strength of about 86 MPa. It was selected for its widespread industrial use and relevance to the study’s objective of improving flexural strength through the incorporation of surface-modified nanoclay. The nanoclay employed in this study is a surface-modified montmorillonite nanoclay, produced by Sigma-Aldrich (St. Louis, MO, USA), with a composition of 34–45% *w*/*w* dimethyl dialkyl amine. The average particle size of this powdered nanoclay is less than 20 microns, while the bulk density ranges from 200 to 500 kg/m^3^. During the measurements, an organic peroxide catalyst and a wax-based release agent were also used. The former is Methyl Ethyl Ketone Peroxide (MEKP) produced by AkzoNobel (Amsterdam, The Netherlands), and the latter is Paste Wax 34D, manufactured by Abel Industrie. To evaluate the effect of environmental exposure, the samples were immersed for one week in commercially available buffer solutions with pH values of 4, 7, and 9 produced by Chempur (Piekary Śląskie, Poland).

### 4.2. Synthesis and Modification of Resin Samples

**Figure 9:**
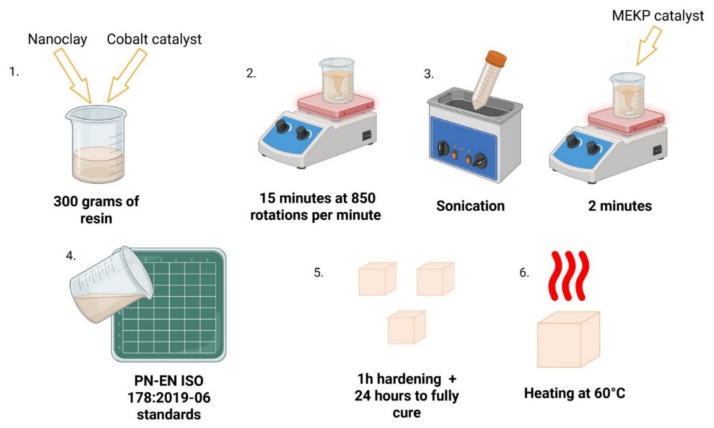
Scheme for obtaining resin modified by the addition of nanoclay. The numbers (1–6) indicate the stages of sample preparation: (1) addition of nanoclay and cobalt octoate catalyst to the resin, (2) mechanical stirring, (3) sonication of the mixture, (4) addition of MEKP hardener and casting into molds, (5) initial curing, and (6) thermal post-curing. Created in BioRender. L. (2026) https://BioRender.com/5mugf85 (accessed o 29 May 2026).

Initially, 300 g of resin was measured and poured into a plastic bucket. The resin was then thermally equilibrated to a constant temperature of 23 °C to ensure uniformity in the subsequent procedures. A catalyst, specifically a 4% Cobalt Octoate solution, was added to the equilibrated resin. The specified amount of nanoclay (ranging from 0.02 wt.% to 1.0 wt.%) was then incorporated into the mixture using a mechanical stirrer for 15 min at 850 rotations per minute (rpm). The mixture was immersed in a water bath at 23 °C and subjected to sonication for 10 min using an ultrasonic cleaner (Ulsonix Cleaning Instruments (Berlin, Germany), PROCLEAN 10.0, 240 W) during the stirring process. Ultrasound was employed to degas the solution and improve the dispersion of nanoclay particles while reducing the formation of agglomerates within the mixture. Following this step, an organic peroxide catalyst (MEKP), which acts as a hardener, was added and mixed for an additional 2 min. Next, the mixture was poured into molds fabricated to meet the dimensions specified by PN-EN ISO 178:2019-06 standards [31], which include 20 sockets measuring 100 mm × 10 mm × 4 mm, within a framework measuring 350 mm × 130 mm × 20 mm. Before use, the molds were covered with a wax-based release agent to facilitate easy removal of the cured samples. In the experiment, degassing was not carried out due to the resin’s short gelation time (approximately 4 min from the addition of peroxide) and because the mold was not closed from the top, allowing the resin to degas naturally during curing. The initial hardening time of the samples was one hour, after which they were removed from the mold and left undisturbed for 24 h to fully cure. Finally, during the post-curing process, the samples were heated in an oven at 60 °C for 1 h under a constant load of 5 kg to ensure dimensional stability and promote complete curing of the polyester matrix. This step ensured the removal of stresses and achieved optimal material properties for the samples. After synthesis, both unmodified samples and those containing nanoclay at concentrations of 0.02, 0.04, 0.06, 0.08, and 0.10 wt.% were subjected to incubation tests to evaluate their chemical resistance. The nanoclay range was selected to evaluate the influence of low filler loadings on the performance of the polyester resin. The objective was to determine whether measurable changes in mechanical and surface properties could be achieved with minimal modification of the matrix composition. The use of low nanoclay concentrations is also advantageous from a practical point of view, as it limits material consumption and preserves the processing characteristics of the resin system. The sample collection procedure is shown in Figure 9.

The samples were immersed in aqueous solutions with pH levels of 9, 7, and 4, simulating different environmental conditions. Each incubation lasted one week, with all samples maintained at room temperature throughout the exposure period.

### 4.3. Fourier Transform Infrared Spectroscopy Analysis

In order to characterize the chemical structure and identify the chemical groups present in the pure resin and composites, Fourier transform infrared spectroscopy (FT-IR) was used. The spectra were collected using a Nicolet iS5 FT-IR spectrometer equipped with an iD7 ATR accessory (Thermo Scientific, Loughborough, UK). Measurements were carried out in the range 4000-400 cm^−1^ at 32 scans per sample and a spectral resolution of 4.0 cm^−1^, at room temperature [32].

### 4.4. X-Ray Diffraction Analysis

In order to determine the phase composition and crystal structure, X-ray diffraction (XRD) analysis was performed for the prepared composites. Diffraction data were collected using an Aeris X-ray diffractometer equipped with a PIXcel1D-Medipix3 detector (Malvern Panalytical, Malvern, UK) and Cu Kα radiation (λ = 1.5406 Å). Measurements were carried out over a 2θ range of 10–70° with a step size of 0.005° and a counting time of 340.425 s per step. The obtained diffraction patterns were used for phase identification and evaluation of the crystallinity of the synthesized composite materials.

### 4.5. Mechanical Characteristics of Resin Samples

To assess the influence of nanoclay content on the mechanical performance of polyester resin-based composites, a series of mechanical tests was conducted, including tensile strength, three-point bending, and impact resistance evaluations. Tensile tests were performed using a Shimadzu AGS-X universal testing machine with a maximum load capacity of 10 kN (Kyoto, Japan). The procedure followed the PN-EN ISO 527 standard [33], applying a crosshead speed of 10 mm/min. Flexural properties were determined using a MTS Criterion 43 universal testing machine (Eden Prairie, MN, USA), operated via MTS TestSuites 1.0 software. The test was conducted in accordance with PN-EN ISO 178, also at a crosshead speed of 10 mm/min. Impact strength was measured using a Zwick/Roell impact testing system (Ulm, Germany). The Charpy method was applied to unnotched samples, with an impact energy of 2 J, in compliance with the PN-EN ISO 179 standard [34]. The hardness of the samples was determined on the Shore D (HD) scale. A Sauter HDD 100-1 hardness tester was used for this purpose, and the thickness of the samples tested was 4 mm. All reported results are presented as mean values ± standard deviation. Tensile, flexural, and hardness measurements were performed using 5 specimens for each material variant. Charpy impact strength measurements were conducted using 10 specimens. Error bars shown in Figure 2, Figure 3, Figure 4, Figure 5, Figure 6 and Figure 7 represent the standard deviation of the measurements.

### 4.6. Surface Morphology and Roughness Analysis

In order to analyze the morphology of the fracture surfaces and evaluate the dispersion of nanoclay within the polymer matrix after tensile testing, scanning electron microscopy (SEM) was conducted. Observations were carried out using a JEOL JSM-IT200 scanning electron microscope (Tokyo, Japan) under low vacuum conditions, at an accelerating voltage of 10 kV, with magnifications of 1000×. Prior to imaging, the fractured composite samples were coated with a thin layer of gold particles using a sputter coater (DII-29030SCTR, JEOL, Tokyo, Japan) to ensure proper conductivity during analysis.

To assess the effect of incubation on surface topography, the average surface roughness (Ra) of the composite samples was measured before and after exposure to different pH environments. For this purpose, a VHX-7000 digital microscope (Keyence International, Mechelen, Belgium) equipped with a 4K CMOS image sensor and the advanced CEO REMAX optical system was used. This high-resolution system allowed for precise, non-destructive surface imaging and roughness profile evaluation. Measurements were conducted to determine changes in surface morphology associated with chemical exposure. The Ra parameter was calculated using the microscope’s dedicated VHX-7000 analysis software, providing a quantitative assessment of surface degradation or modification caused by incubation in solutions with pH 4, 7, and 9.

## 5. Conclusions

This study evaluated the influence of nanoclay modification and environmental exposure on the mechanical and surface properties of polyester-based composites. The incorporation of surface-modified montmorillonite nanoclay affected the material behavior in a complex manner, depending on both the filler concentration and the conditions of chemical incubation. In terms of mechanical performance, nanoclay addition led to improved tensile stiffness (Young’s modulus) prior to incubation, particularly at lower concentrations. However, the influence on flexural strength and impact resistance was less consistent, with certain formulations exhibiting reduced performance, likely due to non-uniform filler dispersion and potential agglomeration. Exposure to acidic, neutral, and alkaline environments significantly altered the mechanical response of the composites, with acidic conditions causing the most pronounced deterioration across all measured parameters. In contrast, some improvements in selected properties were observed after neutral and alkaline incubation, possibly due to secondary crosslinking or surface restructuring. The analysis using SEM supported these findings, revealing differences in fracture surface morphology between unmodified and nanoclay-modified samples. Post-incubation images showed increasingly irregular, brittle fracture patterns, and in some cases visible clusters of filler particles, supporting the hypothesis of microstructural degradation and filler agglomeration. Surface imaging with digital microscopy did not reveal clear morphological changes; however, surface roughness measurements (Ra) confirmed a substantial increase in roughness following incubation, particularly under neutral and alkaline conditions. This suggests that incubation induced degradation or restructuring at the surface level, which may correlate with observed mechanical weakening. A limitation of the present study is that mass change, liquid uptake, and diffusion-related phenomena were not evaluated. Future investigations should include these measurements to provide a more comprehensive understanding of the degradation mechanisms occurring during environmental exposure.

Overall, the results demonstrate that while nanoclay has potential to enhance certain mechanical properties of polyester composites, its effectiveness is highly dependent on filler distribution, environmental conditions, and the targeted property. Future work should focus on optimizing processing parameters to improve nanoclay dispersion and understanding the long-term environmental durability of such systems.

## Figures and Tables

**Table 1:** Comparison of fracture surface morphology of composites exposed to different pH conditions.

Name	Before Incubation	After Incubation at pH 4	After Incubation at pH 7	After Incubation at pH 9
Pure resin	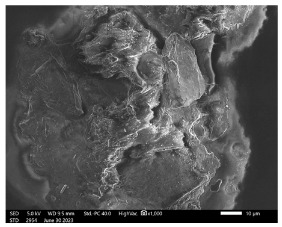	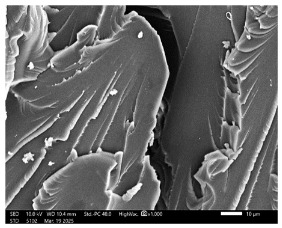	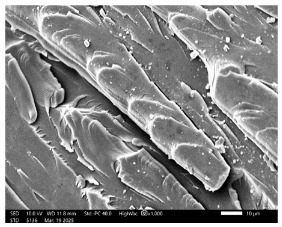	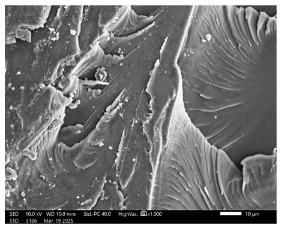
Nc_ 0.02%	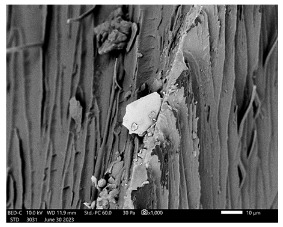	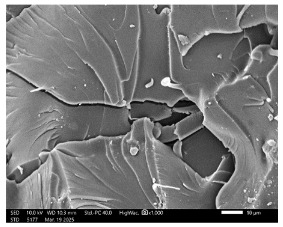	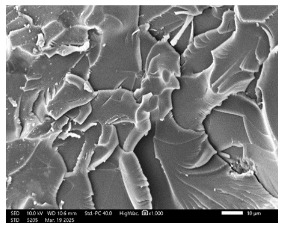	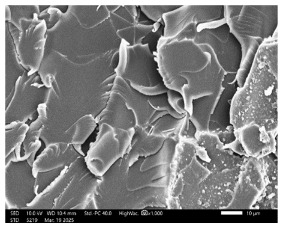
Nc_ 0.04%	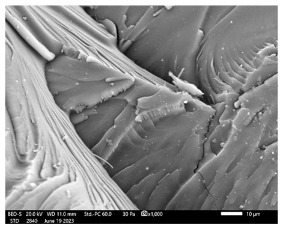	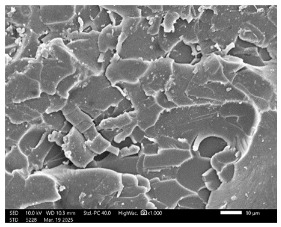	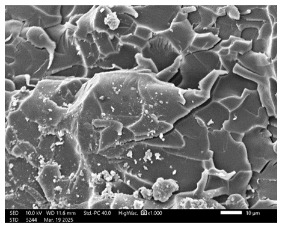	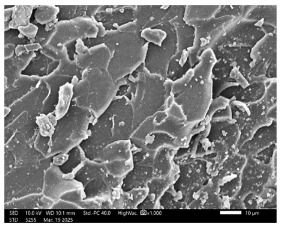
Nc_ 0.06%	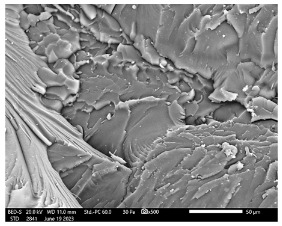	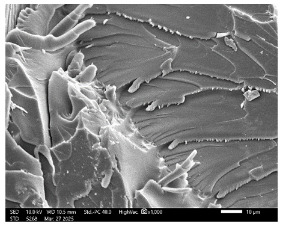	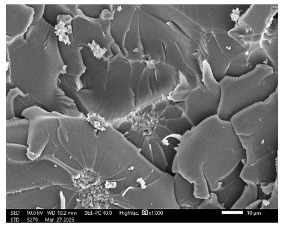	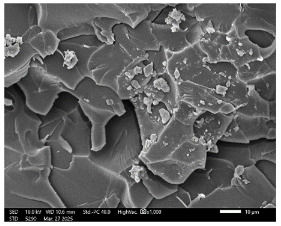
Nc_ 0.08%	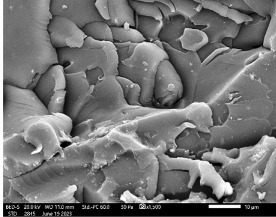	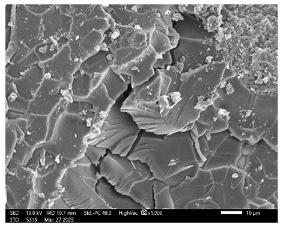	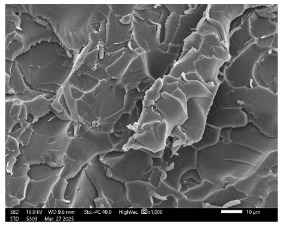	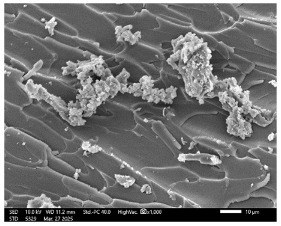

**Table 2:** Digital microscopy images of composite surface morphology before and after incubation.

Name	Before Incubation	After Incubation at pH 4	After Incubation at pH 7	After Incubation at pH 9
Pure resin	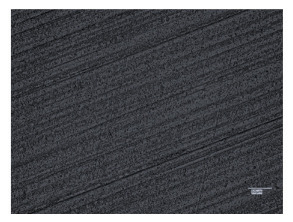	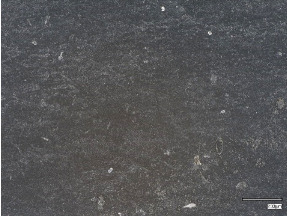	** 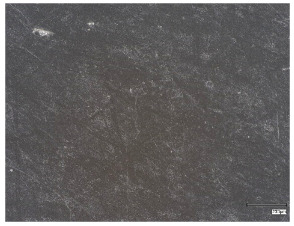 **	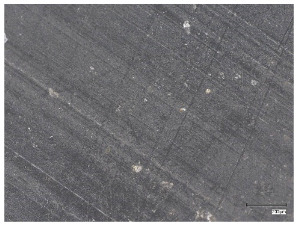
Nc_ 0.02%	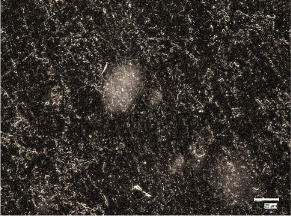	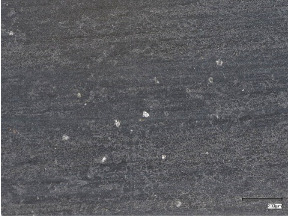	** 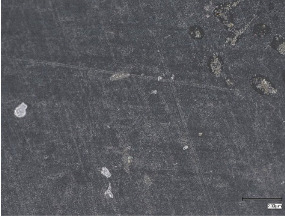 **	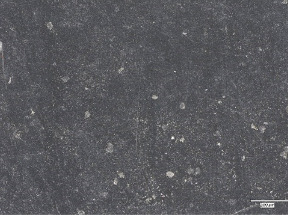
Nc_ 0.04%	** 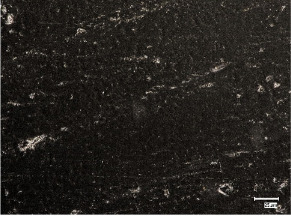 **	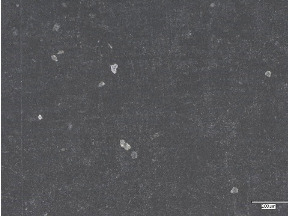	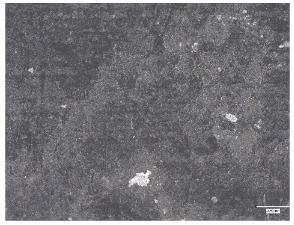	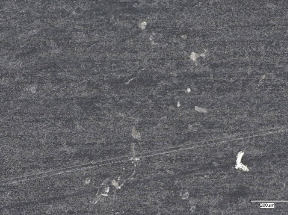
Nc_ 0.06%	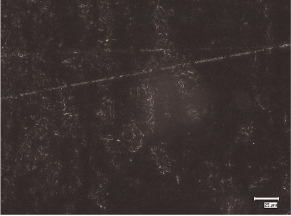	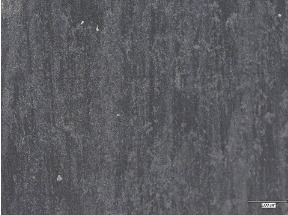	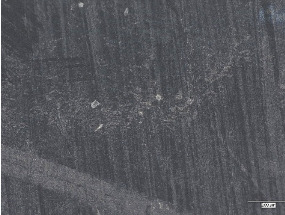	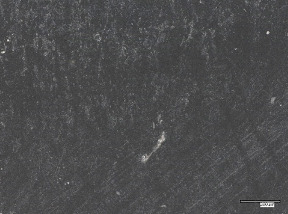
Nc_ 0.08%	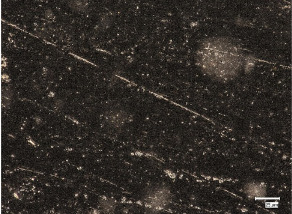	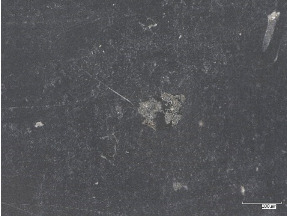	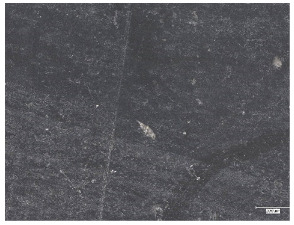	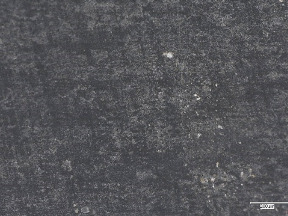

**Table 3:** Average roughness (Ra) of composite samples before and after incubation.

Name	Before Incubation, µm	After Incubation at pH 4, µm	After Incubation at pH 7, µm	After Incubation at pH 9, µm
Pure resin	0.27	0.35	0.30	0.34
Nc-0.02%	0.21	0.44	0.82	0.35
Nc-0.04%	0.24	0.37	0.38	0.43
Nc-0.06%	0.62	0.39	0.25	0.68
Nc-0.08%	0.28	0.45	0.85	0.61

## Data Availability

The raw data supporting the conclusions of this article will be made available by the authors on request.

## Abbreviations

The following abbreviations are used in this manuscript:

FT-IR	Fourier transform infrared spectroscopy
MEKP	Methyl ethyl ketone peroxide
MMT	Montmorillonite
Ra	Surface roughness
SEM	Scanning electron microscopy
